# Interrupted inferior vena cava syndrome discovered incidentally after minimally invasive mitral valve repair in a 31-year-old female patient: A case report

**DOI:** 10.1016/j.ijscr.2023.108621

**Published:** 2023-08-04

**Authors:** Lila H. Abu-Hilal, Duha I. Barghouthi, Tawfiq AbuKeshek, Helmi Tamimi, Hassan Khatib, Abdul-Hakim Dayeh

**Affiliations:** a Faculty of Medicine, Al-Quds University, Jerusalem, Palestine; b Department of Radiology, Al-Makassed Hospital, Jerusalem, Palestine; c Department of Cardiac Surgery, Al-Makassed Hospital, Jerusalem, Palestine

**Keywords:** Inferior vena cava interruption, Minimally invasive cardiac surgery, Femoral cannulation

## Abstract

**Introduction:**

Femoral cannulation is a technique used in minimally invasive cardiac surgery (MICS) for accessing the heart through the femoral artery and vein. However, the presence of an interruption in the inferior vena cava (IVC) can pose challenges during the procedure. Understanding the patient's venous anatomy is crucial to ensure successful cannulation.

**Presentation of case:**

We present the case of a 31-year-old female patient scheduled for minimally invasive mitral valve repair. During the procedure, femoral vein cannulation was unsuccessful. Subsequent diagnostic Computed Tomography (CT) revealed an interrupted IVC with azygos continuation.

**Discussion:**

The interruption of the IVC can make cannulation through the femoral vein difficult or impossible due to the absence of the femoral vein or the presence of a collateral, necessitating alternative approaches. Preoperative imaging, such as CT, plays a significant role in identifying IVC interruptions and guiding surgical planning.

**Conclusion:**

Our case highlights the challenges associated with IVC interruptions during femoral cannulation in MICS. Preoperative imaging is essential for identifying anatomical variations and determining the most appropriate cannulation approach.

## Highlights

• Incidental finding of interrupted IVC during mitral valve repair surgery via minimally invasive technique
• Mitral valve repair via minimally invasive technique in a patient with interrupted IVC and severe mitral regurgitation
• Challenges and modifications in femoral cannulation due to interrupted IVC in MICS
• Anomalous left-sided IVC and successful mitral valve repair in MICS
• Successful conversion to bicaval cannulation in the presence of IVC anomaly during surgery

## 1 Introduction

In MICS, a left or right mini-thoracotomy is used to achieve access to the heart [1]. Femoral cannulation is frequently performed during this procedure to establish extracorporeal circulation [2]. Presence of IVC interruption may lead to difficult or unsuccessful cannulation, so surgeons should be aware of such anomalies prior to surgery [3].

Interruption of the IVC is a congenital anomaly that occurs during embryologic development, and it can result in the formation of various different anomalies. In some cases, this interruption may be associated with azygos or hemiazygos continuation, which can lead to the formation of collaterals that allow for blood flow to bypass the interruption [3].

According to prenatal ultrasound screening, interruption of the IVC with azygos continuation only happens in roughly 1:5000 of the population. It may be related to splenic or cardiac anomalies, although 90 % of the time it is an independent aberration [4]. This particular anomaly involves a disruption of the IVC below the hepatic vein. Consequently, the normal flow of blood above this point is reestablished through the dilation of the azygos and hemiazygos veins, which then empty into the Superior Vena Cava (SVC) [5].

Herein, we present a case of a 31-year-old female patient with interrupted IVC discovered incidentally during minimally invasive mitral valve repair after experiencing difficulty advancing the venous cannula inserted in the right femoral vein.

This case has been reported in line with the SCARE criteria [11].

## 2 Case presentation

We present the case of a 31-year-old woman who initially sought medical attention during her pregnancy ten years ago due to complaints of palpitations. Echocardiography performed at that time revealed mild mitral valve regurgitation. She remained under regular follow-up with her cardiologist and was asymptomatic until two months prior to her admission when she began experiencing palpitations accompanied by shortness of breath on minimal exertion.There were no symptoms related to IVC anomaly.

**Fig. 1:**
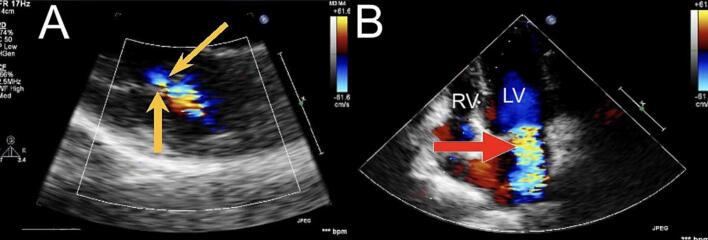
Pre-operative two-dimensional echocardiogram demonstrating severe mitral regurgitation. (A) Long-axis view showing a 0.7 cm vena contracta indicated by yellow arrows. (B) Apical four chamber view showing regurgitant flow (red arrow) indicating severe mitral stenosis. RV: right ventricle, LV: left ventricle. (For interpretation of the references to color in this figure legend, the reader is referred to the web version of this article.)

Physical examination revealed a holosystolic murmur over the mitral area, normal sinus rhythm on electrocardiography, and normal chest X-ray findings. Transthoracic echocardiography demonstrated severe mitral regurgitation with preserved ventricular function and no additional abnormalities (Fig. 1).

As a result, she was advised to undergo mitral valve repair via minimally invasive technique. During surgery, femoral cannulation was attempted to attach the Cardiopulmonary Bypass machine via the femoral artery and vein respectively. Insertion of the arterial cannula went smoothly. However, there was difficulty advancing the femoral vein cannulation.

**Fig. 2:**
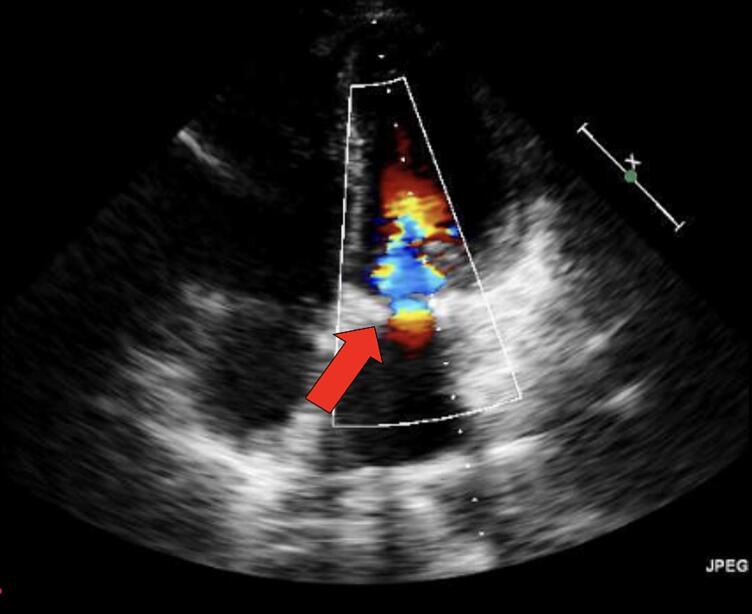
Post operative - four chamber view shows no mitral regurgitation (red arrow). (For interpretation of the references to color in this figure legend, the reader is referred to the web version of this article.)

Transesophageal Echocardiogram (TEE) was done in the operation room. On bicaval view, the guidewire was seen in the right atrium and cannula was inserted after, but it was not visible. It was assumed that the cannula was too short, so a longer cannula was introduced and despite multiple efforts, the cannula was still not seen on TEE and venous cannulation was unsuccessful. The approach was then converted to bicaval cannulation via the same thoracotomy incision and the procedure went smoothly. Postoperative course was uneventful and post mitral valve repair echocardiography showed good left ventricular function and repaired mitral valve, with no regurgitation or stenosis (Fig. 2).

**Fig. 3:**
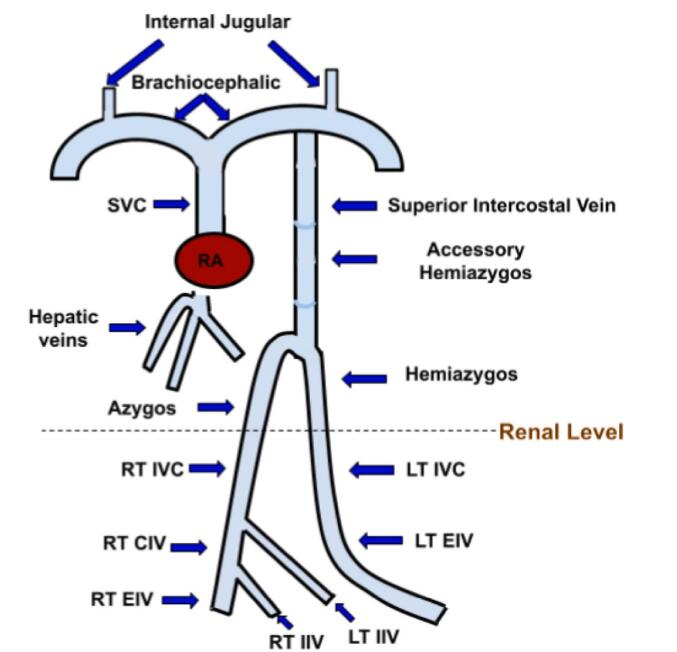
Schematic representation of anomalous venous anatomy in our patient. CIV: common iliac vein; EIV: external iliac vein; IIV: internal iliac vein; IVC: inferior vena cava; LT: left; RT: right; RA: right atrium; SVC: superior vena cava.

**Fig. 4:**
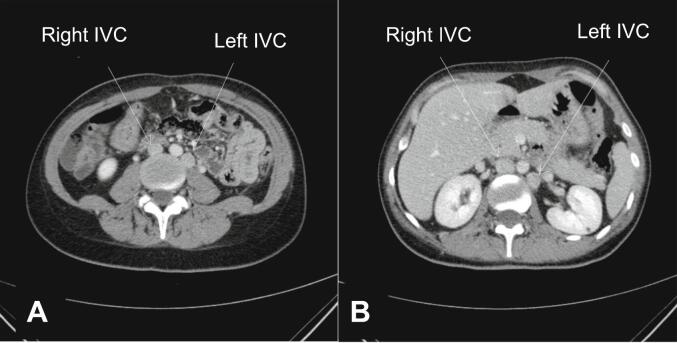
Axial images of contrast enhanced CT (portal phase) shows duplicated IVC at infra-renal (A) and renal levels (B).

**Fig. 5:**
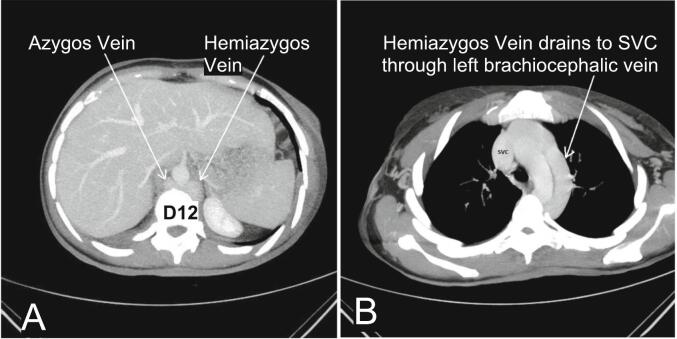
Axial maximum intensity projection (MIP) images of contrast-enhanced CT (portal phase) demonstrate the convergence of the azygos-hemiazygos system at the level of the D12 vertebra (A). Subsequently, the hemiazygos vein drains into the SVC through the left brachiocephalic vein (B).

Post-surgery, a comprehensive examination was conducted to investigate IVC anomalies due to the failed femoral vein cannulation. Abdomen-pelvic CT with IV contrast revealed duplicated IVC with interconnection through the left internal iliac vein. The infra-hepatic segment of IVC was absent, and the hepatic veins directly adjoined the right atrium. Both right and left IVC continued as azygos and hemiazygos vein, respectively. The azygos and hemiazygos veins converged at the level of the D12 vertebral body and drained through the left brachiocephalic vein and SVC (Fig. 3, Fig. 4, Fig. 5).

Following successful mitral valve repair surgery, the patient underwent postoperative follow-up to ensure her well-being. The incidental finding of an interrupted IVC was communicated to the patient for future reference. However, as she remained asymptomatic without complications or hemodynamic issues, no specific intervention or treatment was necessary. The patient was reassured that this information would be documented in her medical records for future medical interventions. Regular follow-up appointments were scheduled to monitor her cardiovascular health and assess post-surgery recovery. At the six-month mark, the patient was completely asymptomatic. She was advised to promptly report any new symptoms or concerns.

## 3 Discussion

Femoral cannulation is a technique used in MICS that involves accessing the heart through the femoral artery and vein. However, in some cases, the patient may have an interruption of the IVC, which can complicate the procedure. Preoperative imaging, such as CT, can be used to confirm safe femoral cannulation [2].

The development of the IVC in embryology occurs during the fifth to seventh weeks of gestation. It is a complex process involving the formation, regression, and fusion of the postcardinal, subcardinal, and supracardinal veins [6]. This results in the formation of the five embryological structures comprising the IVC: iliac, subrenal, renal, suprarenal segment, and hepatic segments [7,8]. Interruptions in this process can cause anomalies, including segment agenesis or fusion of the suprarenal and hepatic segments [7]. These anomalies have clinical implications, such as deep vein thrombosis (DVT) or varicose veins, and require proper identification for diagnosis and treatment.

Multidetector row computed tomography (MDCT) and magnetic resonance imaging (MRI), are reliable non-invasive techniques for identifying IVC anomalies, accurately depicting the anatomy and variations of the IVC, and detecting any associated anomalies [3]. Additionally, physical examination and patient history, including polysplenia or a wide mediastinum, can also offer clues to IVC anomalies [2].

Patients with interrupted IVC and azygos or hemiazygos continuation may be asymptomatic, while those without such continuation or collateral drainage may experience symptoms like DVT or varicose veins. Proper diagnosis and treatment, including anticoagulants or surgery, are needed to prevent complications. Surgical intervention may be required for severe IVC anomalies without azygos continuation [3].

Anomalous regression of the right supracardinal vein and persistence of the left supracardinal vein can result in a rare anatomical variation known as a left-sided IVC, occurring in about 0.2 % to 0.5 % of cases. In this variation, the bilateral common iliac veins drain into the left-sided IVC, which typically courses upward to join the left renal vein. Notably, deviations from this typical configuration can occur, such as the presence of a hemiazygos continuation or a retroaortic right renal vein. These variations highlight the complex nature of vascular anatomy and underscore the importance of comprehensive diagnostic assessment when encountering atypical venous patterns [9].

In addition to femoral cannulation, other cannulation techniques in MICS can provide alternative options. Options for arterial cannulation in MICS include the ascending aorta, femoral artery, or axillary artery [10]. These alternatives may be considered in cases where femoral artery cannulation fails, as in our case. Preoperative computed tomography angiography (CTA) can play a significant role in providing vital information about arterial stenosis, tortuosity, and aneurysmal disease presence, aiding in the selection of an appropriate cannulation site. CTA is recommended before MICS procedures, especially with femoral arterial cannulation, as it offers correlation with surface anatomy and assists in precise incision planning [10].

Our case report highlights the challenges and need for modifications in the presence of IVC anatomical variations. Further studies should evaluate intra-operative imaging techniques such as conventional venogram or abdominal ultrasound, and pre-operative screening for IVC variations, considering feasibility, cost-effectiveness, and limitations. Surgeons should adopt a systematic approach, incorporating intra-operative imaging and alternative cannulation techniques. Although no preoperative findings indicated an IVC anomaly in our patient, emphasizing the potential for such variations is essential. Future research using advanced imaging modalities like MDCT or MRI may enhance preoperative detection of IVC anomalies and improve patient selection for MICS.

Case reports contribute to knowledge and guide decision-making, but further research is necessary for optimal management of IVC anatomical variations in cardiac surgery.

## 4 Conclusion

This case report highlights the importance of recognizing and accommodating IVC anomalies in MICS. Surgeons should adjust cannulation strategies for procedural success and safety, maintaining a high index of suspicion for IVC anomalies. Further research and sharing similar cases are crucial for expanding the literature and improving patient care in MICS.

## Ethical approval

This case report was conducted at Al-Makassed Hospital, and it is important to note that the study is exempt from ethical approval in our institution. The exemption was granted based on the nature of the study, which involves a single case report and does not involve experimental interventions or Manuscript without author details additional procedures beyond routine clinical care. Patient approval was obtained for the publication of this case report, and all necessary measures were taken to ensure patient confidentiality and privacy.

## Funding

This case report received no specific grant from any funding agency in the public, commercial, or not-for-profit sectors.

## Credit authorship contribution statement

Study Concept or design: Lila H. Abu-Hilal, Duha I. Barghouthi

Writing the manuscript: Lila H. Abu-Hilal, Duha I. Barghouthi, Tawfiq AbuKeshek

Review and editing the manuscript: Helmi Tamimi, Hassan Khatib, Abdul- Hakim Dayeh

## Guarantor

Dr. Hassan Khatib.

## Methods

The work has been reported in line with the SCARE criteria [11].

## Patient consent

Written informed consent was obtained from the patient for publication of this case report and accompanying images. A copy of the written consent is available for review by the Editor-in-Chief of this journal on request.

## Declaration of competing interest

The authors declare that the research was conducted in the absence of any commercial or financial relationships that could be construed as a potential conflict of interest.

## Data Availability

The original contributions presented in this study are included in this article/supplementary material, further inquiries can be directed to the corresponding authors.
